# Differential diagnosis in chronic psychosis: a case report

**DOI:** 10.1192/j.eurpsy.2024.1511

**Published:** 2024-08-27

**Authors:** C. Alcalde-Diosdado Crespi, M. D. P. Almodóvar Rivera, F. Vílchez Español

**Affiliations:** Hospital Universitario de Jaén, Jaén, Spain

## Abstract

**Introduction:**

A colombian woman aged 62, with previous diagnosis of paranoid schizophrenia. She retired from working 2 years ago, when she started taking care of her sick husband full-time.

**Objectives:**

To describe a chronic psychosis case and discuss the treatment options and differential diagnosis.

**Methods:**

We used the face-to-face interviews during her last hospitalization and her electronic medical history.

We also made a brief research about the effectiveness of risperidone depot in Pubmed.

**Results:**

**Psychiatric history**

She’s had 3 hospitalizations, all of them coincided with stressful vital situations. The first one occurred when she was dealing with a job issue. In the second one she was having an economic conflict with her husband. And the third one has coincided with worries about her retirement pension and her caregiver burden.

**Current episode**

She came to my hospital emergency department distressed because she thought her husband and her were victims of an international drug trafficking plot. She said a colombian drug cartel had sent 9 prostitutes to her village in order to steal from them, by pretending they were cleaning assistants, as a reprisal against her husband, who used to be a military in Colombia . She explained the nature of this event with great details. Also, she said the electric company was involved and they had tried to intoxicate her.

The psychopathological exploration was altered with a correct speech in its form but incoherent in its content. She presented a highly structured delusional plot of prosecution. No major affective disorders were detected. She suffered from reactive insomnia and anxiety.

**Evolution**

At first, it was torpid, she felt perspicacious and angry about the admission. Later, as the antipsychotic started to work, the symptoms improved and she became calm and collaborative. She has never criticized the delusion plot, but it was encapsulated, and the affective resonance disappeared.

We tried oral aripiprazole and paliperidone but retired both due to bad tolerance. Then we tried risperidone, with good tolerance and response, and started her current monthly treatment with Risperidone 100 mg depot.

After one year, she has kept stable with no relapses and good adherence to the treatment.

**Differential diagnosis**

The patient meets every diagnostic criteria of Delusional disorder (F22), as she has never experienced hallucinations and the functionality has not significantly decreased through the years. Also, she doesn’t meet the second criteria for Schizophrenia (F20).

**Conclusions:**

It is important to explore the evolution of a psychotic disorder in order to differentiate between a schizophrenia and a delusional disorder, as the prognosis differs significantly.

Using Risperidone monthly depot can be a good option for treating a psychotic disorder (Sampson et al. Cochrane Database Syst Rev 2016. 14;4(4)).

**Disclosure of Interest:**

None Declared

